# Decoding chronic pain: integrating genetics, neuroimaging, and AI for precision management

**DOI:** 10.3389/fpain.2026.1747942

**Published:** 2026-02-06

**Authors:** Bade Uckac, Natalia S. Ogonowski, Luis M. García-Marín, Santiago Diaz-Torres, Scott F. Farrell, Dale R. Nyholt, Miguel E. Rentería

**Affiliations:** 1 Brain and Mental Health Program, QIMR Berghofer Medical Research Institute, Brisbane, QLD, Australia; 2 School of Biomedical Sciences, Faculty of Health, Queensland University of Technology, Brisbane, QLD, Australia; 3 School of Biomedical Sciences, Faculty of Health, Medicine and Behavioural Sciences (HMBS), The University of Queensland, Brisbane, QLD, Australia; 4 Population Health Program, QIMR Berghofer Medical Research Institute, Brisbane, QLD, Australia; 5 RECOVER Injury Research Centre & NHMRC Centre for Research Excellence in Better Health Outcomes for Compensable Injury, The University of Queensland, Brisbane, QLD, Australia; 6 Centre for Innovation in Pain and Health Research, The University of Queensland, Brisbane, QLD, Australia; 7 STARS Education and Research Alliance, Surgical Treatment and Rehabilitation Service (STARS), The University of Queensland and Metro North Health, Brisbane, QLD, Australia

**Keywords:** chronic pain, clinical management, epidemiology, genetics, machine learning, neuroimaging

## Abstract

Chronic pain is increasingly recognised as a standalone medical condition shaped by interacting biological, psychological, and social determinants, affecting nearly one in three adults worldwide. This review synthesises contemporary evidence on the epidemiology, mechanisms, and management of chronic pain, with emphasis on the convergence of genetic, neurobiological, and psychosocial factors. We draw on recent population-based studies, clinical trials, neuroimaging research, and multi-omic genetic analyses to highlight the complexity and heterogeneity of this condition. Chronic pain disproportionately affects older adults, women, and socioeconomically disadvantaged groups, and frequently co-occurs with psychiatric, cardiovascular, and neurodegenerative disorders, reflecting shared pathways of maladaptive neuroplasticity. Although pharmacological therapies often provide modest long-term benefit, integrated psychological, physiotherapeutic, and interventional approaches demonstrate more sustainable improvements in function and quality of life. Advances in genomics and large-scale genome-wide association studies (GWAS) have revealed extensive polygenic overlap with psychiatric and immune traits, while neuroimaging consistently demonstrates alterations within prefrontal, insular, and limbic circuits that shape pain perception and persistence. Despite reliance on subjective symptom reporting, emerging digital phenotyping, wearables, and AI tools offer promising avenues for objective monitoring and personalised treatment. Integrating biological, behavioural, and environmental data will be essential to achieving truly precision-based chronic pain care.

## Introduction

Chronic pain is a high-impact medical disorder arising from the interaction of biological, psychological, and social processes. It affects an estimated 30%–33% of the global population, including individuals in low- and middle-income countries, and represents a major contributor to disability and healthcare burden worldwide (1, 2). The International Classification of Diseases, 11th Revision (ICD-11), developed by the World Health Organisation (WHO), defines chronic pain as pain that persists or recurs for longer than three months (3). This framework distinguishes between chronic primary pain, in which pain itself constitutes the primary health condition, and chronic secondary pain, in which pain arises as a symptom of an underlying disease. Optional extension codes further characterise pain severity across the intensity, interference, and distress domains, each rated on a 0–10 scale using instruments such as the Numerical Rating Scale (NRS) or the Visual Analogue Scale (VAS). Temporal patterns are also specified, including continuous or episodic pain (4). Within this framework, pain is increasingly described using mechanistic descriptors. Nociceptive pain arises from actual or threatened tissue damage and activation of peripheral nociceptors, typically associated with inflammation or mechanical injury (5). Neuropathic pain results from a lesion or disease affecting the somatosensory nervous system (6). Nociplastic pain refers to pain arising from altered central nociceptive processing in the absence of clear tissue damage or somatosensory system pathology, and is commonly associated with central sensitisation and disrupted pain modulation (7).

Chronic pain imposes a major and uneven public health burden. In the United States, 20.9% of adults report chronic pain, and 6.9% experience high-impact pain associated with substantial disability (8). Sex differences emerge after puberty and persist across the lifespan, with women generally reporting greater pain sensitivity and fluctuations linked to hormonal and social influences (9). Prevalence increases steeply with age, from ∼14% in young adults to more than 60% in those ≥75 years, reflecting the combined effects of ageing, multimorbidity, and social determinants of health (10). Transition from acute to chronic pain is common, with estimates approaching 80%–90% in some settings (11). Approximately one-third of chronic postsurgical or post-traumatic pain cases have neuropathic features, highlighting the need for accurate phenotyping and tailored care pathways (12).

**Figure 1:**
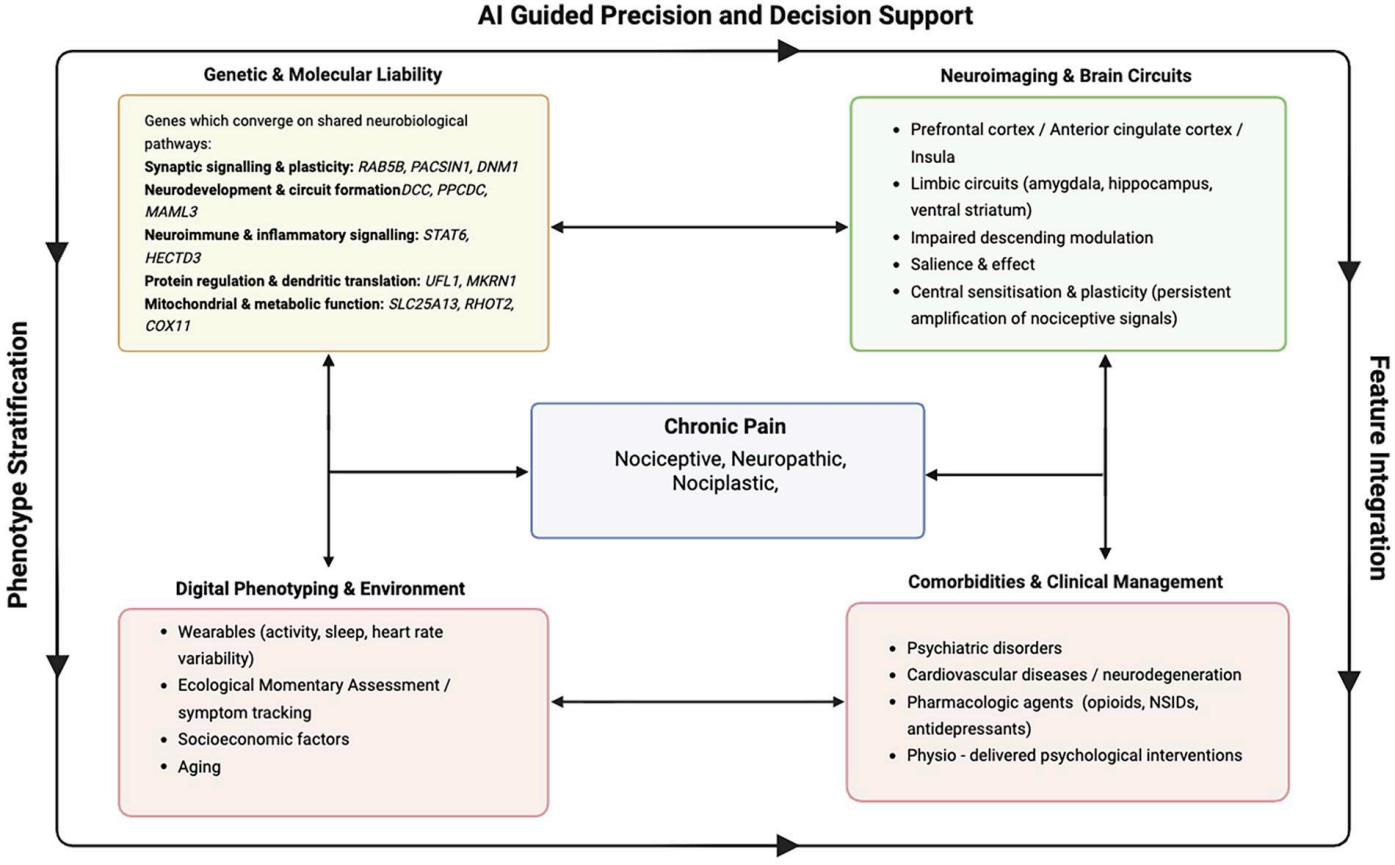
Conceptual precision framework for chronic pain. This figure depicts how genetic and molecular liability, neuroimaging-derived brain circuit alterations, digital phenotyping and environmental exposures, and comorbidities and clinical management jointly inform the characterisation of chronic pain. These domains feed into AI-guided precision and decision support, with phenotype stratification and feature integration as overarching processes that organise and synthesise multimodal data to support personalised chronic pain care.

Advances in neuroimaging, genetics, and clinical research increasingly show that chronic pain reflects interactions between environmental exposures (e.g., occupational strain, inactivity, socioeconomic disadvantage) and co-occurring psychological, cardiovascular, and neurodegenerative conditions (Figure 1). These insights support a standardised biopsychosocial framework that improves clinical assessment and research stratification.

Despite these advances in mechanistic understanding, the clinical management of chronic pain is still confounded by its heterogeneity, overlapping comorbidities, insufficient diagnostic capacities, and lack of application of research methods into the practice. First-line pharmacotherapeutic agents [e.g., nonsteroidal anti-inflammatory drugs (NSAIDs), antidepressants, gabapentinoids, and opioids], are mostly preferred to mitigate the chronic pain and can reduce symptoms in selected patients, but benefits are often small to moderate and frequently offset by toxicity, tolerance, dependence risk, or poor long-term adherence. Those widely used analgesics primarily target peripheral nociceptive or neurotransmitter pathways and do not reliably address central sensitisation and maladaptive neuroplasticity that maintain persistent pain (13).

This review provides a multidisciplinary overview of chronic pain, including epidemiology, comorbidities, genetic risk, brain mechanisms, and therapeutic approaches. We examine population-level burden and disparities, highlight shared biological pathways underlying comorbidity, and summarise genetic and neuroimaging evidence for heritable risk and altered brain circuitry. Pharmacological, interventional, and non-pharmacological strategies are discussed within a translational framework. Finally, we evaluate emerging machine-learning and AI-enabled tools for improving phenotyping, prognosis, and personalised management in chronic pain.

## Methods

We employed a narrative synthesis approach in this review, focusing on studies published in the past 5 years. We consulted PubMed and Scopus, using initial terms including “*chronic pain*” or “*multisite chronic pain*” and either “*polygenic*”, “*genetics*”, “*GWAS*”, “*machine learning*”, “*AI*”, “*artificial intelligence*”, “*wearable*”, and “*precision medicine*”. We prioritised primary research articles, meta-analyses, and clinical trials, with additional sources identified through citation chaining.

## Results

### Chronic pain measurement: wearables

Wearable devices are increasingly used in pain research and management to provide objective, continuous assessment of pain-related physiology and behaviour. Activity trackers and biosensors capture indices such as heart rate variability (HRV), physical activity, sleep, and stress (14). These markers reflect underlying pain processes: reduced HRV indicates autonomic dysregulation with heightened sympathetic tone, while disturbed sleep and restricted movement correlate with greater pain severity and functional impairment (15). By monitoring real-time fluctuations, wearables characterise the dynamic nature of pain beyond self-report. When combined with machine-learning approaches, multimodal sensor data may also support the prediction of pain exacerbations and treatment response (16). However, most wearable-derived signals are indirect correlates of pain and lack biological specificity for pain mechanisms. Data quality can also be affected by sensor drift, missing data, contextual confounding (other medical illnesses, stress, medication changes), and variable user adherence, which may introduce systematic bias if dropout or non-wear time correlates with symptom severity. These limitations underscore the need for robust preprocessing, transparent data reporting, and validation against clinically meaningful endpoints.

### Comorbidities and overlapping pathophysiology

Chronic pain rarely occurs in isolation and most commonly coexists with mental health disorders, which represent some of its most frequent and clinically significant comorbidities (17). These relationships are bidirectional and likely mediated by shared neural circuitry involving the prefrontal cortex, anterior cingulate, and amygdala, which regulate both affect and pain perception (18). Furthermore, the presence of other psychiatric conditions such as post-traumatic stress disorder (PTSD), panic disorder, and generalised anxiety disorder (GAD) is associated with up to 60% of the cases. Similarly, early-life consultations for emotional distress or psychological symptoms, often historically referred to as “nerves”, are associated with a significantly increased likelihood of seeking medical care for long-term pain in later life (19, 20).

Cardiovascular disease also occurs disproportionately among people with chronic pain. A large Taiwanese cohort (∼17,000 participants) reported a 20% increased risk of myocardial infarction and 30% increased risk of stroke compared with pain-free controls, independent of traditional risk factors (21). Another study showed that genetic liability to multisite chronic pain (MCP), pain in more than two body sites for over three months, is associated with increased risk of major adverse cardiac and cerebrovascular events (MACCE), including myocardial infarction and stroke (22). This is supported by studies showing increased levels of pro- inflammatory cytokines and autonomic nervous system dysfunction among patients (23).

Chronic pain has further been associated with accelerated cognitive decline, particularly memory impairment from around age 60 (24, 25). In dementia care facilities, 54.6%–78.6% of residents report pain across subtypes, and up to 45.8% of individuals with Alzheimer's disease report persistent pain. Neuroimaging and animal studies have identified structural and functional changes in cognition-related brain regions in response to most non- specific chronic neuropathic and inflammatory pain (26). However, more localised phenotypes, such as chronic musculoskeletal pain and headaches, remain underexplored in this context (27). These findings reinforce that chronic pain is a multisystem condition embedded within and contributing to a broader cycle of comorbidity and declining health.

### Genetic approaches and polygenic architecture

Genetic studies demonstrate that chronic pain has a complex polygenic architecture shaped by common and rare variants across neurological, immune, and psychological pathways (28). Twin and family studies estimate heritability to be between ∼7%–59%, depending on the phenotype and study design (29). Earlier GWAS of multisite chronic pain (MCP) in the UK Biobank identified 76 associated loci, and a subsequent meta-analysis expanded this to 343 independent loci, including 92 novel signals (30). Sex-stratified analyses revealed marked differences, with 115 loci in males and 12 in females. Male-specific signals are implicated in synaptic and neurotransmitter pathways (e.g., NXPH2, SNCA, DAAM1), whereas female-specific loci (e.g., MAML3, GABRB2) are related to neural development and GABAergic signalling. Multi-omic integration highlighted genes such as WDR90 and DNM1, which are enriched for functions in sleep regulation, substance use, and synaptic vesicle cycling (31, 32). Pathway analyses implicated synaptic function, neuronal development, and axon guidance, while colocalisation identified 43 potentially causal genes expressed across brain tissues, including HECTD3, CCDC17, DNM1, PCDHA1, and WDR90 (33). MCP also showed pleiotropic and likely causal relationships with psychiatric and substance-use disorders, cortical-thickness variation, and >11,000 shared causal variants with traits including insomnia, MDD, PTSD, neuroticism, and opioid use (34).

Polygenic risk scores (PRS) (35) and Mendelian randomisation (MR) (36) enable translation of GWAS findings into population-level inference. PRSs for overlapping chronic pain conditions predict centralised pain burden (37), although phenotype-specific prediction remains modest (e.g., chronic back pain AUC ≈ 0.56) (37). MR studies indicate that genetic liability to MCP increases risk of cardiometabolic, psychiatric, and autoimmune outcomes (38, 39), while vitamin D may influence depression more strongly than chronic pain (40). These approaches require rigorous attention to ancestry bias, instrument strength, and pleiotropy, and results should be interpreted alongside epidemiology and biological evidence before informing clinical practice. Reporting PRS risk to patients remains debated but may support future clinical stratification (41).

Integrative multi-omic studies have further identified high-confidence causal genes regulating brain protein abundance, including *DCC* (dopaminergic development), *PPCDC* (coenzyme A synthesis), and ubiquitination-related regulators *UFL1* and *MKRN1* (42, 43). Additional loci influence synaptic plasticity (*RAB5B, STAT6, PACSIN1)*, mitochondrial and metabolic function (*SLC44A2, C15orf57, SLC25A13*), and neuronal survival (*RHOT2, COX11, RMDN3, PCMT1*) (44–46). Together, these findings link genetic variation to neural circuitry, inflammation, metabolism, and neuroplasticity, providing a foundation for precision therapeutics supported by experimental validation.

### The potential of neuroimaging

Neuroimaging has reshaped pain research by revealing reproducible structural and functional brain changes associated with the persistence and heterogeneity of chronic pain. Consistent findings include reduced insular grey matter and altered prefrontal–frontoparietal connectivity in chronic low back pain, together with aberrant medial prefrontal activity on resting-state analyses (47, 48). Machine-learning studies further show that connectivity and cerebral blood-flow patterns across thalamic, prefrontal, posterior cingulate, and insular–somatosensory circuits correlate with pain intensity, suggesting potential biomarkers, although current evidence remains exploratory and requires harmonised protocols, replication, and larger samples (49).

Large-scale initiatives such as ENIGMA now pool MRI and rs-fMRI data from >70 cohorts, enabling robust detection of shared and condition-specific brain correlates of chronic pain (50). Early findings indicate widespread variation in cortical thickness, subcortical volume, and salience-network connectivity across pain disorders. Population-scale analyses similarly reveal overlapping but distinct neuroanatomical signatures across musculoskeletal, headache, and visceral pain, with the ventromedial prefrontal cortex emerging as a hub linking pain to negative affect and suicidality (51).

Electrophysiology provides complementary insight. EEG-derived features, such as peak alpha frequency and δ/θ spectral activity, may predict vulnerability to chronic pain and distinguish individuals who transition from acute to persistent pain (52). Experimental modulation of central pain signatures (e.g., SIIPS-1) further supports causal brain contributions (53, 54). While clinically immature, these advances point toward biologically grounded patient stratification.

Functional-connectivity (FC) clustering in migraine without aura has identified distinct subgroups characterised by thalamic, hippocampal, and amygdalar alterations, illustrating the potential for circuit-targeted therapies (55). Overall, convergent neuroimaging, EEG, and computational approaches position brain-network dysfunction as a core feature of chronic pain and a foundation for biomarker development, though high cost, methodological variability, and limited clinical validation highlight the need for multinational, well-funded collaboration to achieve translation.

### Chronic pain management: beyond medications

Pharmacotherapeutic agents such as opioids, antidepressants, and NSAIDs remain first-line and accessible options for chronic pain management (56). Opioids dampen nociceptive transmission via µ-opioid receptor pathways; antidepressants/gabapentinoids modulate monoaminergic or calcium-channel–linked neurotransmission that can support descending inhibition (57, 58); and NSAIDs primarily reduce peripheral inflammatory mediator signalling. However, these mechanisms may incompletely address central sensitisation, altered salience/affect processing, and cognitive–emotional drivers of disability, helping explain variable effectiveness and discontinuation in long-term use (59). The inconsistent effectiveness and safety concerns associated with pharmacotherapeutic agents led to a shift toward psychological interventions, including cognitive-behavioural therapy (CBT), acceptance and commitment therapy (ACT), and mindfulness-based approaches (57). Their outcomes improve coping, reduce disability, and enhance quality of life, particularly in chronic musculoskeletal pain. Integrating these strategies with physiotherapy has demonstrated meaningful benefits, including improved quality of life, increased self-efficacy in pain management, and reduced pain-catastrophising beliefs (58). A network meta-analysis of 97 randomised controlled trials (RCTs) found that combined psychological-physiotherapy interventions improved pain and function post-intervention, although long-term outcomes remain underexplored. Physiotherapist-delivered psychological interventions consistently outperformed standard care. Positive improvements in pain were observed at short-, medium, and long-term follow-ups, with disability outcomes supporting these interventions across all time points (60). Evidence quality was moderate, and incomplete reporting of interventions limits the reproducibility of these findings.

### Insights from machine learning and artificial intelligence

Machine learning (ML) and artificial intelligence (AI) are increasingly used to develop data-driven tools for chronic pain assessment and treatment planning. ML models can identify predictors of pain severity and treatment response from multimodal datasets integrating physiological, biochemical, demographic, and psychological variables (61, 62). In a retrospective cohort of >11,000 patients, random forest and neural-network models achieved >85% accuracy in predicting pain relief, although validation and prospective testing remain essential to avoid overestimation (63).

Large language models (LLMs) can analyse patient narratives, such as fibromyalgia reports, reducing clinician workload while maintaining diagnostic accuracy (64). Emerging clinical decision-support systems combining LLMs with retrieval-augmented generation (RAG) integrate patient data with medical evidence to deliver tailored recommendations for conditions such as chronic low back pain (65). Multimodal frameworks that combine BERT-based analysis of symptom questionnaires with EEG-derived image features (ResNet50) have also classified neuropathic pain severity with ∼60% accuracy, demonstrating the potential of jointly modelling textual and physiological data (66).

Data-driven clustering approaches further elucidate heterogeneity in chronic pain. Unsupervised ML applied to broad symptom profiles has revealed reproducible biotypes reflecting graded psychosocial burden, while similar analyses in clinical cohorts identify subgroups distinguished by psychiatric comorbidity and functional impairment, with distress-dominant phenotypes showing poorer outcomes (67). Multimodal clustering integrating neuroimaging, psychosocial variables, and physical function has likewise identified distinct back-pain subtypes (68). Nonetheless, most ML/AI studies in chronic pain remain exploratory, with performance often optimised on single datasets. Key barriers to translation include dataset shift across sites/devices, limited external validation, unclear model interpretability, and inadequate clinical actionability of predicted risk scores or clusters. Prospective evaluation and reporting standards that enable reproducibility will be essential before near-term deployment in routine care.

## Discussion

Chronic pain remains underrecognised in research and policy, partly due to ongoing debate over whether it represents a symptom of underlying pathology or a disease with its own maladaptive neurobiology. This ambiguity has hindered investment, biomarker discovery, and coordinated care pathways. Progress requires integrated translational frameworks linking genetic, neural, physiological, behavioural, and environmental determinants to patient-centred outcomes. While ICD-11 definitions and self-report tools provide essential structure, their value is greatly enhanced by continuous multimodal phenotyping (e.g., wearables, HRV, sleep, actigraphy, momentary assessment) that captures symptom dynamics, identifies physiologically distinct subgroups, and improves characterisation of treatment-response variability (48).

Genomic and multi-omic evidence shows chronic pain is highly polygenic, involving synaptic, mitochondrial, neuroimmune, and neuroplasticity pathways. Translation of these findings requires caution regarding ancestry bias, small effects, and premature clinical application. Neuroimaging and electrophysiology consistently demonstrate alterations in prefrontal, insular, limbic, thalamocortical, and striatal circuits, reflecting dysregulated nociceptive, affective, and cognitive processing. However, biomarker development is constrained by small or convenience samples, heterogeneous methods, limited replication, and predominantly cross-sectional designs. Diagnostic heterogeneity, comorbid depression, and medication effects further confound interpretation, even within large consortia such as ENIGMA.

Machine-learning and AI approaches offer scalable tools to integrate multimodal data, predict pain trajectories, define mechanistic subtypes, and support personalised interventions (69). Yet clinical translation depends on transparent models, explainability, ethical data governance, and rigorous external validation to prevent bias. Heterogeneity in feature engineering and evaluation frameworks currently limits reproducibility and synthesis, and deep-learning models often lack interpretability for clinical use.

### Future directions

To date, genetic, neuroimaging, and digital phenotyping studies of chronic pain have largely progressed in parallel rather than within truly integrated frameworks. Each modality captures a distinct yet complementary aspect of chronic pain biology: genomic data index stable, lifelong susceptibility; neuroimaging reflects intermediate, circuit-level alterations in nociceptive, affective, and cognitive processing; and wearable or ecological data capture dynamic, state-dependent symptom expression in daily life. While single-modality models, such as PRS-based stratification or imaging-derived signatures, offer advantages in interpretability and feasibility, they are limited in their ability to capture interactions across biological scales or to account for temporal variability in pain experience. Multimodal fusion approaches hold promise for overcoming these limitations by integrating complementary sources of information, but they introduce additional challenges related to data harmonisation, missing data, differing cofounders across studies, and interpretability. Addressing these trade-offs through transparent benchmarking and reporting in diversity across ancestry, sex, socioeconomic status, and life stages is essential. Ethical considerations of research, including equitable sampling, responsible communication of results, and robust data privacy protections, should remain integral to technological advancement.

Ultimately, translation into practice will require strong clinician–researcher partnerships, improved genomic and data-science literacy within health systems, and alignment with regulatory and ethical frameworks. Coordinated efforts across academia, healthcare, industry, and patient advocacy will be essential to ensure that emerging precision approaches deliver equitable and meaningful benefits for individuals living with chronic pain.
